# Cognitive Reflection and the Diligent Worker: An Experimental Study of Millennials

**DOI:** 10.1371/journal.pone.0141243

**Published:** 2015-11-06

**Authors:** Brice Corgnet, Roberto Hernán Gonzalez, Ricardo Mateo

**Affiliations:** 1 George L. Argyros School of Business and Economics, Chapman University, Orange, California, United States of America; 2 Division of Industrial Economics and Finance, Business School, University of Nottingham, Nottingham, Nottinghamshire, United Kingdom; 3 Business Department, Universidad de Navarra, Pamplona, Navarra, Spain; University of the Basque Country, SPAIN

## Abstract

Recent studies have shown that despite crucially needing the creative talent of *millennials* (people born after 1980) organizations have been reluctant to hire young workers because of their supposed lack of diligence. We propose to help resolve this dilemma by studying the determinants of task performance and shirking behaviors of *millennials* in a laboratory work environment. We find that cognitive ability is a good predictor of task performance in line with previous literature. In contrast with previous research, personality traits do not consistently predict either task performance or shirking behaviors. Shirking behaviors, as measured by the time participants spent browsing the internet for non-work purposes (*Cyberloafing*), were only explained by the performance on the Cognitive Reflection Test (CRT). This finding echoes recent research in cognitive psychology according to which conventional measures of cognitive ability only assess a narrow concept of rational thinking (the algorithmic mind) that fails to capture individuals’ capacity to reflect and control their impulses. Our findings suggest that hiring diligent *millennials* relies on the use of novel cognitive measures such as CRT in lieu of standard personality and intelligence tests.

## Introduction

“*I never could have done what I have done without the habits of punctuality*, *order*, *and diligence*, *without the determination to concentrate myself on one subject at a time*.”

Charles Dickens

A study conducted in the summer of 2012 reveals that managers selected from a representative sample of US industries were significantly more inclined to hire senior workers than *millennials* for the same level of experience [1]. Managers’ inclination for seniors was mostly explained by their higher level of reliability and professionalism compared to younger workers. Given that *millennials* represent the workforce of the future, their supposed lack of diligence poses a growing challenge to organizations.

“*The challenge for employers with this generation is providing an environment that values the balanced life while encouraging millennials to be diligent workers as well*.”

([2] page 133)

Managers recognized, however, the growing need for young workers’ skills such as a creative mind and an acute understanding of new technologies. The hiring dilemma of *millennials* motivated our study of the relationship between diligent work behavior, cognitive abilities and personality traits among young adults. To assess diligence on the job, we measured task performance by recording productivity and accuracy on the work task as well as shirking behavior of participant workers in a newly developed virtual workplace. Shirking was assessed by the amount of time workers spent browsing the internet for non-work purposes instead of completing their assigned task (*Cyberloafing*). The time spent on the internet for non-work purposes directly detracts workers from their task, costing money to the organization [3]. [4] estimates that *cyberloafing* costs U.S. corporations at least $85 billion each year. Our measure of diligent work behavior includes both task performance and shirking behaviors which closely relate to two important dimensions of overall work performance: core task performance and counterproductive work behaviors [5][6]. Core job performance is typically assessed using objective measures such as productivity on the job. Counterproductive work behaviors refer to behaviors that harm the well-being of the organization or its members, such as theft, carelessness at work, sexual harassment, *cyberloafing* or misuse of resources and information.

### Cognitive measures, task performance and cyberloafing

Intelligence (as measured, for example, by IQ or academic tests such as the Scholastic Aptitude Test -SAT-) has been found to be the main predictor of job performance. The correlation between intelligence tests and work performance is about 0.84, when using objective measures of performance, and ranges from 0.56 to 0.74, when using supervisors’ ratings [7][8][9]. Intelligence measures have been found to consistently predict job performance in a wide variety of occupations (see [10] for a review) ranging from military to civilian jobs and across multiple sectors and job categories (e.g. [11][12]). As a result, we expect measures of cognitive ability to correlate positively to performance on the work task.

**Hypothesis 1**: *We expect a positive relationship between cognitive ability and task performance*.

**Fig 1:**
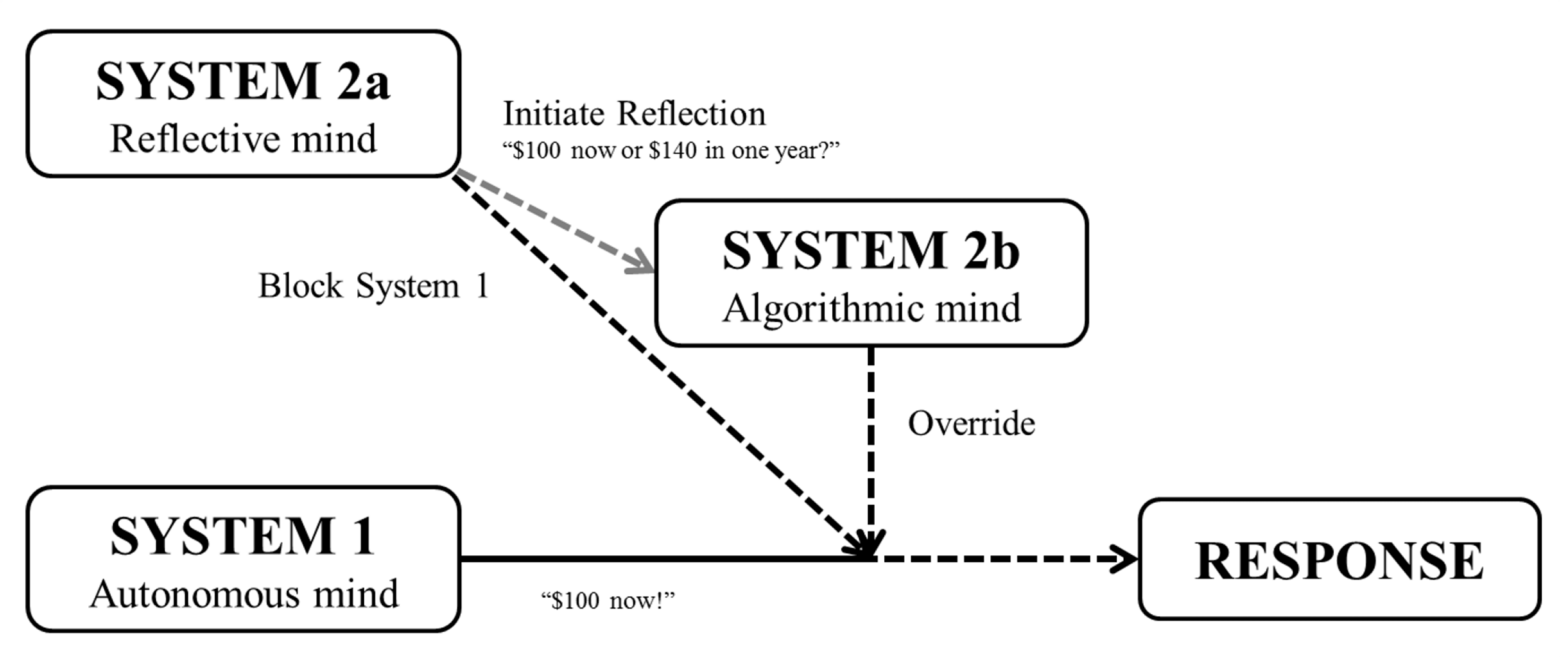
Tripartite model (Stanovich, 2009).

Regarding *cyberloafing*, we build on the work of [13] which shows that cognitive ability, as measured by participants test scores on the Shipley Institute of Living Scale, is negatively correlated with objective measures of counterproductive work behaviors (e.g., destruction of property, physical violence) (ρ = -.21 and ρ = -.36, respectively). The authors also find other indirect measures of cognitive ability, such as college grades or years of education, to correlate negatively, although mildly, to counterproductive work behaviors. Some studies have failed to report a negative relationship between intelligence measures and counterproductive work behaviors when using self-reported measures of counterproductive work behaviors [14][15][16][17]. The negative relationship between counterproductive work behaviors and intelligence may be due to the inhibitory effect of intelligence with regard to deviant behaviors as is conjectured by [13]. This conjecture is in line with Jensen’s argument that: “*persons with low IQ fail to adequately and realistically imagine the future consequences of their actions*. *Their immediate behavior is therefore less thoughtful and more impulsive*” ([18], p. 572). Additionally, [18] shows that the inability to delay gratification, which has been shown to correlate negatively with general measures of intelligence and working memory [19][20], relates to higher incidence of deviant behaviors in adults. Regarding *cyberloafing*, we conjecture that people with high cognitive ability will more easily refrain from browsing the web for immediate gratification to keep working on the task for later payments (at the end of the experiment). Therefore, we expect a negative relationship between cognitive ability and *cyberloafing*. One cognitive measure known as the Cognitive Reflection Test (CRT, henceforth) has been specifically designed to capture cognitive impulsiveness and was found to correlate to delay gratification more significantly than other cognitive measures including SAT [21]. The CRT consists of three questions which all have an appealing and intuitive, yet incorrect, answer (see S2 Text). Upon reflection, one can disregard the intuitive answer and find the correct one. We thus expect the negative relationship between cognitive ability and *cyberloafing* to be more pronounced for CRT than for the other measures of cognitive skills. This conjecture also follows from the tripartite model developed by [22][23] which stresses the distinction between algorithmic and reflective processes. Algorithmic processes which are measured with standard intelligence tests (e.g. SAT) are typically associated with computational efficiency whereas reflective processes which can be measured with the CRT [24] are associated with critical and rational thought. This tripartite model is an extension of the dual systems model (e.g [25] for a review) where a response to a stimulus can either be quick and automatic (System 1) or effortful and deliberative (System 2). In Fig 1, we illustrate the tripartite model of [22][23] using the delayed gratification question that consists in choosing between $100 now and $140 in one year from now [21]. The automatic mind (System 1) is unreflective and provides an effortless response “*Grab the $100 now*!” which may eventually be blocked and reassessed by the reflective mind (System 2a) “*Could waiting to get more money ($140) be a better answer*?”. The final response is computed by System 2b (algorithmic mind) after assessing the appeal of delayed gratification. To do so, the algorithmic mind will be calculating either explicitly or implicitly the present value of $140 in one year from now. After completing these computations, System 2b will override the automatic response of System 1 if the discounted value of the future payment is greater than the immediate payment. System 2b is distinct from System 2a as it relies on computational capacity and does not engage, unlike System 2a, in reflection to block System 1 automatic response. Consistently with the tripartite model, [21] reports that the capacity to delay gratification is less correlated with general measures of intelligence (e.g. SAT and Wonderlic test: System 2b) than with CRT scores (System 2a).

In our organizational setup, one may think of browsing the internet (for example to check one’s *Facebook* page) as immediate gratification (System 1 response) so that refraining from doing so will crucially hinges upon one’s capacity to block this automatic response and stay focused on the work task (System 2a). This reflective strategy will lead to delayed gratification in the form of increased task earnings which will be collected at the end of the experiment.

Based on the previous arguments, we consider the following conjecture.

**Hypothesis 2**: *We expect a negative relationship between cognitive ability and cyberloafing*. *We expect this negative relationship to be more pronounced for CRT than for the other measures of cognitive ability*.

### Personality, task performance and cyberloafing

The relationship between personality and job performance has been studied at length in the last decades (e.g. [26][27][28][29][30]). This literature has focused on five fundamental dimensions of personality which are commonly referred to as the “Big Five personality traits” [31][32][33][34][35]. The Big Five personality traits include the following dimensions: extraversion, agreeableness, conscientiousness, emotional stability (the opposite of neuroticism), and openness. Among these five personality traits, conscientiousness and emotional stability have been the most consistent predictors of job performance across a variety of tasks and occupations [27][36][37][38][39][40][41][42][43][44]. Conscientiousness is a broad personality dimension defined as the extent to which a person is able to self-regulate and be purposeful, achievement oriented, responsible, and persistent [32]. Individuals with high levels of conscientiousness are described as organized, reliable, and ambitious [35][45]. They also are skilled planners who maintain impulse control (e.g., act cautiously, delay gratification, and follow rules and norms), which often leads to enhanced task performance [45]. Individuals with low levels of emotional stability are insecure, nervous, lack self-esteem, and doubt their abilities. This lack of confidence and the incapacity to cope with stress affects on-the-job performance negatively. It follows that people with high levels of conscientiousness and emotional stability exhibit high levels of task performance [46]. The other personality traits (extraversion, agreeableness, and openness) have not been found to consistently predict job performance [30]. Based on these previous findings, we conjecture that conscientiousness and emotional stability will affect task performance positively.

**Hypothesis 3**: *We expect conscientiousness and emotional stability to relate positively to task performance*. *We do not expect the other personality traits to affect task performance*.

Conscientiousness and emotional stability have also been found to relate negatively to counterproductive work behaviors [47]. In addition, integrity tests based on conscientiousness, emotional stability and openness have been found to predict behaviors such as sabotaging products or equipment, theft, alcohol or drug abuse as well as being belligerent with customers and co-workers [29]. Based on these findings, we conjecture that conscientiousness, emotional ability and to a lesser extent openness will relate to *cyberloafing* negatively.

**Hypothesis 4**: *We expect conscientiousness*, *emotional stability and openness to relate negatively to cyberloafing*. *We do not expect the other personality traits to affect cyberloafing*.

## Materials and Methods

Chapman University IRB committee approved this research. All participants in the experiments provided an electronic signature on the consent form by signing into the database to participate in the experiments. No deception was used.

### Participants

Participants were 264 undergraduates (52.27% male; average age 20.08) enrolled in a pool of potential participants at a Western U.S. university. All participants were born between 1981 and 2000 which corresponds to the standard definition of the *millennials* generation [2]. They responded to an email offering $15 for a one-hour survey. These participants were recruited from a pool of 426 people (49.05% male; average age 20.20) who participated in an organizational study three months earlier. In the organizational study, participants were responding to an email offering $7 plus an unspecified amount of bonus money for participation in an experiment lasting 2.5 hours earning an average of $26.60.

We report no significant differences between the 264 participants who came back for the survey and the 162 who did not regarding our different measures of diligent work behavior (Mann-Whitney-Wilcoxon tests–*MWW*-, *MWW* = -1.000, *p-value* = 0.32 for productivity; *MWW* = -0.375, *p-value =* 0.499 for accuracy; and *MWW* = 1.514, *p-value =* 0.13 for *cyberloafing*). Also, age and gender composition did not significantly differ between the two samples (*p-values* > 0.5 for both the Mann-Whitney-Wilcoxon test and the proportion test).

### Design of the organizational study

In the organizational study which was used to recruit the people for the survey, participants were randomly assigned to one of seven organizational settings which differed in the type of incentives (individual or team incentives), the availability of communication (chat) and the possibility for participants to monitor each other’s activities (watch). Each organizational setting included a work task and internet access. In Table 1, we summarize the seven organizational settings according to its main features.

**Table 1:** Main features of the organizational settings.

Organizational Setting	Incentives	Chat^a^	Watch^a^	Number of observations
1	Team	Peers	Top-down	47
2	Team	No	Top-down	41
3	Team	Peers	No	38
4	Team	No	No	30
5	Individual	No	No	37
6	Team	No	Peers	38
7	Team	Peers	Peers	33

^a^In the “peers” conditions, subjects could communicate with, or watch what other workers were doing. In the “top-down” conditions only one subject in the session (in the role of the boss) could watch others’ activities.

In this paper, we focus on the relationship between personal characteristics (personality traits and cognitive abilities) and work behavior letting aside the analysis of organizational features on work performance (see [48][49]).

The instructions indicated that participants could choose among several activities. Participants could spend as much or as little time as they wanted on the various activities, each of which was undertaken on a separate screen. To switch activities, participants simply had to choose the corresponding option from an action menu at the bottom of their screens. We describe each of these activities below.

#### The Work Task

**Fig 2:**
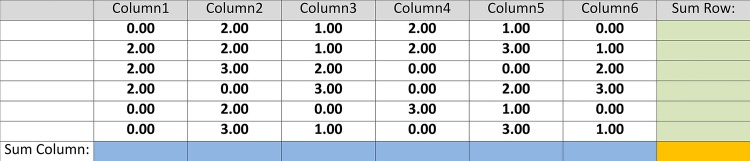
Work task.

Adapted from previous research using summation tasks [50], we implemented a particularly long and laborious task intended to resemble the monotony that can accompany organizational life and prompt *cyberloafing*. The task required participants to sum up tables of 36 numbers without using a pen, scratch paper, or calculator (see Fig 2). Each table had six rows and six columns of randomly-generated integers between zero and ten. Before providing the grand total in the bottom-right cell, participants had to provide a separate subtotal for the 12 rows and columns. Calculating these subtotals did not directly generate earnings but could help participants compute the grand total, which generated a 40-cent profit if correct and a 20-cent penalty if incorrect. After completing a table, participants learned whether their answer was correct and how much money they earned. At the end of each period, participants learned the total amount of money generated by all ten participants.

#### Internet

If participants chose internet, the work task window was replaced by an internet window which was embedded in the software. Within the bounds of university policy, participants could use the internet however they liked, including sending and receiving email. Their confidentiality was assured and maintained, but the software did track the exact amount of time spent on each activity. Although participants could not complete the work task while browsing the internet, switching was quick and easy. By clicking the relevant button, participants returned to either the last internet page or the last number table that they had seen.

#### Monitoring

In four of the seven organizational settings which were considered in this study, participants could also monitor the activities of other workers in the organization. In two of these conditions only one subject in the session (in the role of the boss) could watch others’ activities. If participants selected the monitoring option from the action menu, they used a separate screen to choose whom to monitor (anywhere from one to all other participants). For each selected participant, a column in a table listed their activities (e.g., switched to internet, provided a subtotal), their current earnings, and their percentage contribution to the firm total production. Participants who were being monitored saw an eye picture and a text message indicating that “[Monitor’s experiment ID] is watching you”.

#### Communication

In three of the seven organizational settings, participants could exchange instant messages with other workers. This virtual form of communication was chosen to maintain anonymity. Participants who chose to communicate entered a chat room in which they could send a message to one or more people.

#### Instruction Period

Upon arrival at the lab, participants were seated at private computer terminals and began to read instructions on their computers. They had 20 minutes to read the instructions, with a timer displayed on a large screen at the front of the lab. The instructions indicated that they were one of ten members of a virtual firm; the members of the firm would undertake a 1-hour and 40-minute task, broken up into 20-minute periods. Each member would work on the task, separately and in isolation. The bonus would be calculated based on firm performance (an equal share of total production for each of the firm members), except for one organizational setting (individual incentives) in which participants were rewarded based on their own performance.

We provide a summary of the instructions for the baseline setting without monitoring and communication in S1 Text.

### Design of the survey

In the survey, participants were asked to answer a series of questions regarding demographics and personality traits as well as complete a series of tasks measuring their cognitive skills. Participants were paid a participation fee of $15 in addition to which they could obtain lottery tickets by successfully completing an adding task. A prize of $400 was paid to a single winner among all survey participants.

#### Measures for the organizational study

*Productivity* was defined as the total number of summations completed in the experiment (*M* = 31.85, *SD* = 13.70).

*Accuracy* was defined as the percentage of correct tables with respect to the total number of tables completed (*M* = 67.44%, *SD* = 26.70%).

*Cyberloafing* was defined as the percentage of a participant’s total time that was spent on the internet screen (*M* = 10.28%, *SD* = 17.13%). The time spent on the internet was considered shirking since it was time away from the work task for which consulting the internet was pointless. We confirm this interpretation of internet usage in our organizational setting in [51] in which we show, for example, that allowing internet access to participants reduce productivity by close to 20%. Also, we show that a worker’s accuracy on the summation task decreases right after spending time browsing the web. In line with these previous findings, we find *cyberloafing* to be negatively correlated to both productivity (ρ = -.433, *p-value* < 0.001) and accuracy (ρ = -.331, *p-value* < 0.001).

### Measures for the survey

We provide a complete description of the survey, including descriptive statistics of the different measures in S2 Text.

#### Demographics

We asked participants their name, gender as well as parents’ education levels. We also asked participants how many hours a week they usually work for pay (Work experience) or volunteer (Volunteering). We also collected data regarding the number of hours participants usually spend on the internet in a given day (Internet habits). We asked participants which school they were attending. We constructed a dummy for each school which takes value one if a student belongs to a given school (e.g. Humanities and Social Sciences, Business and Economics, Science) and value zero otherwise. We assessed religiosity by asking participants how often they attended religious services. We finally asked participants to report their last math grade in High School as a control for math skills.

#### Cognitive measures

We measured cognitive reflection using the CRT developed by [21] (*M* = 1.22, *SD* = 1.10). The distribution of CRT scores in our sample does not significantly differ from the distribution reported by [21] which was based on a sample of 3,428 respondents (χ^2^(3) = 3.606, *p-value* = 0.307). We collected (self-reported) information regarding grade point average (GPA) (*M* = 3.43, *SD* = 0.38) as well as SAT scores (*M* = 1,861.87, *SD* = 242.72). These scores do not significantly differ from the average GPA (3.49) and SAT (1,807) of students at the school where the experiments were conducted (*p-values* > 0.5). We also assessed participants’ adding skills by asking them to sum five one-digit numbers for a duration of five minutes following [52]. This task was incentivized using a lottery mechanism. For each correct answer, participants received one lottery ticket to win a single prize of $400 across all participants. We constructed the *Adding skills* variable as the number of correct answers in the five-minute task (*M* = 28.94, *SD* = 9.68). This variable provides a measure for participants’ skills on the work task of the organizational study as well as a correlate for working memory [53] which is seen as an important dimension of general intelligence [54]. Similarly to [21], we observe low to moderate positive correlation across cognitive measures (see Table 2). In line with Frederick’s study, CRT and SAT correlate positively although moderately (*r* = 0.35) which suggests that SAT and CRT are not entirely measuring the same cognitive skills [22][23]. Also, note that even though GPA and SAT are significantly correlated, no such correlation exists between GPA and CRT.

**Table 2:** Correlation matrix for cognitive measures.

	CRT	GPA	SAT	ADDING SKILLS
CRT	1			
GPA	0.0402	1		
SAT	0.3534****	0.1990***	1	
ADDING SKILLS	0.2614****	0.0232	0.1726***	1

*** p-value < .01

**** p-value < .001

#### Personality traits

We also conducted a personality test to assess the five dimensions of personality: extraversion, agreeableness, conscientiousness, neuroticism and openness [55]. We used the 44-item version of this test which was developed by [56] and [57]. The Cronbach’s alpha reliability scores for extraversion, agreeableness, conscientiousness, neuroticism and openness were 0.87, 0.78, 0.80, 0.81 and 0.80, respectively.

#### Organizational features

In our analysis, we control for the organizational features which characterized the virtual workplace environment in which participants were involved in the organizational study. We define three organizational features (Incentive schemes, Chat and Watch) and construct a dummy variable for each feature. The incentives dummy takes value one if a survey participant was involved in an organization in which members were paid according to individual incentives. This dummy variable takes value zero when organizational members were paid a team bonus (an equal share of total production for each of the firm members). The Chat (Watch) dummy variable takes value one if survey participants had access to communication (monitoring) in the organization in which they were involved. The Chat (Watch) dummy takes value zero otherwise.

## Results and Discussion

We conduct regression analyses to assess the significance of demographic factors, cognitive measures as well as personality traits on diligent behavior at work. We consider productivity, accuracy and *cyberloafing* as dependent variables.

We use OLS regressions with robust standard errors and clusters at the session level [58]. In line with hypothesis 1, we find that *Adding skills*, SAT and CRT affect positively productivity and accuracy on the work task (see Tables A and B in S3 Text). Overall, cognitive measures have a positive impact on task performance with the exception of GPA. Regarding demographics, the only variable which has a significant and positive effect on task performance across regressions is work experience. We summarize our results as follows.

**Result 1 (Task performance and cognitive measures).** *We report a positive and statistically significant relationship between cognitive measures (Adding skills*, *CRT and SAT) and task performance*, *with the exception of GPA*.

Regarding *cyberloafing*, we show that CRT is the only cognitive measure that significantly predicts internet usage on the job (see Table C in S3 Text). This result is consistent with the conjecture stated in hypothesis 2 according to which the negative relationship between cognitive ability and *cyberloafing* is expected to be more pronounced for CRT than for the other cognitive measures.

**Result 2 (Cyberloafing and cognitive measures).** *We report a negative and statistically significant relationship between CRT and cyberloafing*. *The other cognitive measures (Adding skills*, *SAT and GPA) do not significantly affect cyberloafing*.

More generally, our findings are consistent with recent research [22][23] emphasizing a crucial distinction between algorithmic and reflective processes. Our findings show that algorithmic skills predict task performance while not being suited for a broader assessment of work behavior. On the contrary, reflective skills appear to be essential for predicting both task performance and shirking behaviors on the job. A recent study correlating collegiate success and cognitive skills also appears to be consistent with our findings [59]. The authors show that timely graduation as well as graduation within six years are best predicted by a measure of backward induction (the “Hit 15” test) and not by IQ tests. The “Hit 15” test is a measure of cognitive ability which is distinct from IQ and which resembles CRT as it requires substantial reflection [60].

In line with hypothesis 3, productivity and accuracy on the task appear to be unrelated to the following personality traits: extraversion, agreeableness and openness. In contrast to hypothesis 3, we find no significant relationship between neuroticism and task performance (see Table A in S3 Text). In line with hypothesis 3, conscientiousness affects accuracy positively, although this relationship is only marginally significant (see Table B in S3 Text). In line with hypothesis 4, conscientiousness reduces *cyberloafing*, although only mildly. Emotional stability and openness also relate negatively to *cyberloafing* but these relationships are not statistically significant (see Table C in S3 Text). We summarize these results below.

**Result 3 (Task performance, cyberloafing and personality traits).** *We report a positive (but marginally significant) relationship between conscientiousness and accuracy and a negative (but marginally significant) relationship between conscientiousness and cyberloafing*. *The other personality traits do not affect work behavior*.

**Fig 3:**
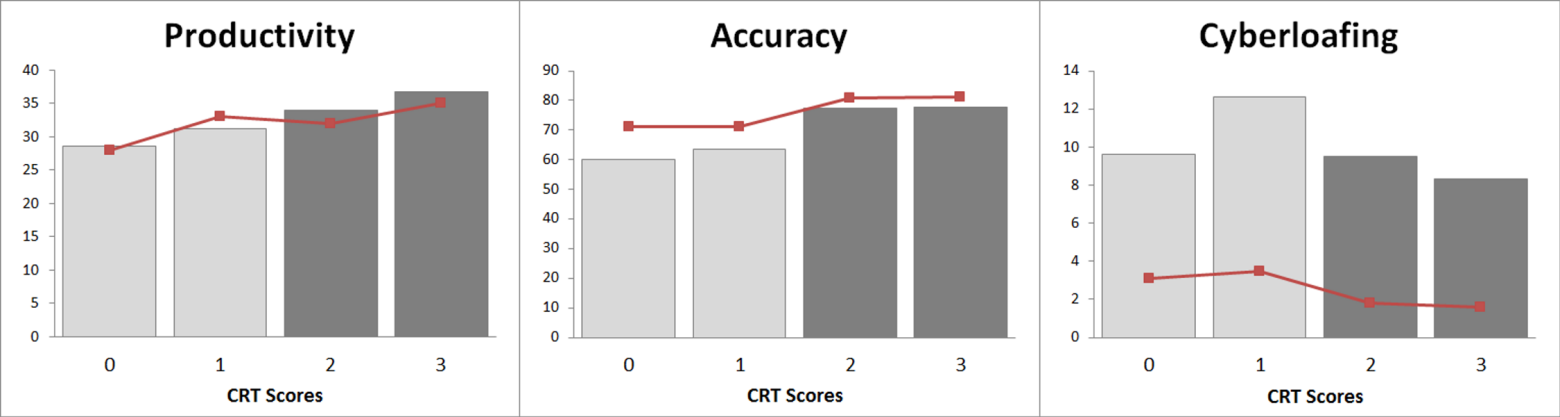
Performance and CRT. Average *productivity* (left panel), *accuracy* (middle panel), and *cyberloafing* (right panel) across CRT scores (median values represented with brown line).

As an intuitive overview of our findings we represent productivity, accuracy and *cyberloafing* as a function of CRT scores. We observe that productivity and accuracy are increasing with CRT scores whereas *cyberloafing* tends to decrease with CRT scores (see Fig 3). Using standard parametric and non-parametric tests, we confirm that productivity and accuracy increase with CRT scores whereas cyberloafing decreases (see S4 Text).

In S4 Text, we also analyze the effect of CRT scores on *productivity*, *accuracy* and *cyberloafing* for each of the seven organizational settings (as described in Table 1) separately. It should not be surprising that, despite pointing in the same direction as the regression analyses with pooled data, the reported effect of CRT scores fail to reach significance in a number of cases, which could be due to the relatively small numbers of observations for each of the organizational setting taken separately (between 30 and 47 observations). Given the low number of observations per organizational setting we do not think the current study is suited to assess a possible interaction effect between CRT scores and organizational settings features. Furthermore, personal characteristics (e.g. gender or studies) may have a strong effect when the sample size is small, hiding the relationship between cognitive abilities and subjects performance. We nonetheless conjecture that CRT scores and personal characteristics in general are expected to have a lesser effect in explaining work behavior in work environments in which monetary incentives are strong.

## Conclusions

We developed a novel approach to the analysis of work behavior by using a virtual workplace environment which allows us to precisely assess job performance and shirking behaviors on the job. Beyond the methodological novelty of our analysis, we also contributed to the current literature by stressing the importance of the distinction between standard measures of cognitive abilities (e.g. SAT and GPA) and measures of cognitive reflection (e.g. CRT) in understanding work behavior. Our results provide a clear recommendation to recruiters hiring *millennials*: include cognitive reflection tests to your conventional battery of tests. Another potential advantage of recognizing the importance of cognitive reflection over conventional intelligence tests is that, unlike IQ scores which have been found to be relatively stable overtime (e.g. [61]), workers could perhaps be trained to be more reflective.

Finally, our findings call for a general assessment of the role of rational thinking [24] on organizational behavior. For example, future research may attempt to study the link between broad measures of rationality and the capacity to solve problems and innovate.

## Supporting Information

S1 Text Organizational study. Instructions.
(DOCX)

S2 Text Survey description. (DOCX)

S3 Text Regression results. (DOCX)

S4 Text Descriptive statistics and standard tests. (DOCX)
