# Supplementary material for: Cognitive Reflection and the Diligent Worker: An Experimental Study of Millennials
Source: PLoS One. 2015 Nov 6;10(11):e0141243. doi: 10.1371/journal.pone.0141243 (PMC4636387; doi:10.1371/journal.pone.0141243)
Supplement: S1 Text — Instructions. (DOCX) [file pone.0141243.s001.docx]

**S1 Text. Organizational study.** Instructions.

Summary of the instructions handed out to the participants in the baseline setting with team incentives and without communication and without monitoring. The complete set of instructions is available at: <https://sites.google.com/site/vodiligentworker/instructions/study-1>.

**Action menu**

**
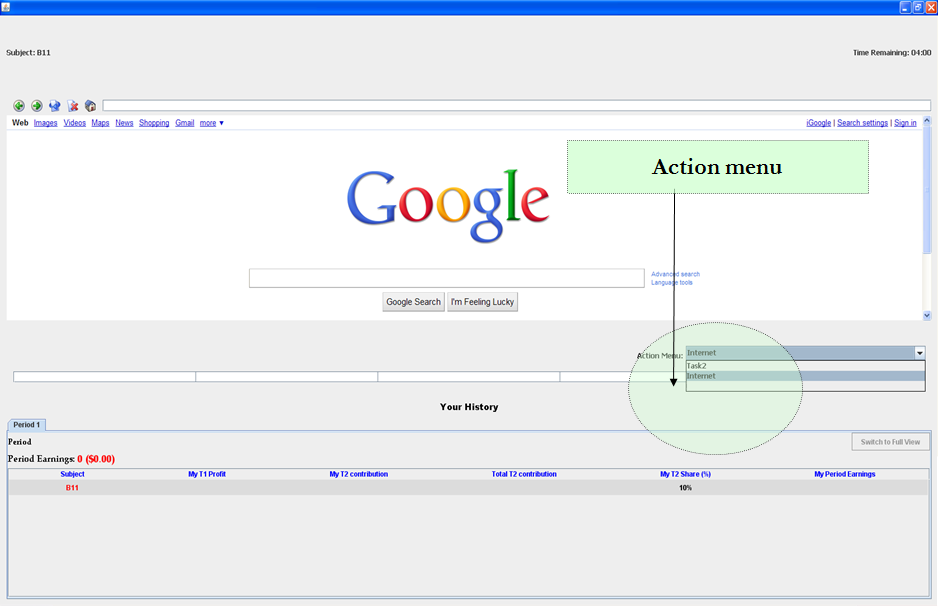
**

**INSTRUCTIONS SUMMARY**

- This experiment involves *10* participants and consists of *5* periods of *20* minutes. In each period you can participate in any of the following *two activities*: *browsing* the internet or *summing up numbers* in a table. To switch from one activity to another you just have to click on the corresponding option of the action menu displayed on your screen. The two activities are referred to as *Task 2* (sum up numbers in a table) and *Internet* (browse the web). Each activity is undertaken separately, in a different screen.

**Task 2 – summing up numbers**

- The amount of money you generate by undertaking *Task 2* is displayed in the third column **“My T2 Contribution”** of the history table at the bottom of the screen.
- The total amount of money generated by all *10* participants in *Task 2* is displayed in the fourth column **“Total T2 Contribution”** of the history table at the bottom of the screen.
- If you sum up the numbers of a table correctly then each participant in the experiment (including yourself) gets *5 cents (10%×50).*
- If you answer *Task 2* incorrectly you generate a penalty of *20 cents* that is subtracted from *Task 2 total contribution*. So when you answer incorrectly your individual earnings (as well as other participants’ earnings) decrease by *2 cents (10%×20). (Your Task 2 earnings can never be less than 0.)*

**Task 1 – yellow box**

- In addition to the previously mentioned activities you can click on a yellow box moving from left to right at the bottom of your screen. This task is referred to as *Task 1*. Your *Task 1 profit* is displayed in the second column of the history table at the bottom of your screen **“My T1 profit”.** Each time you click on the box you make *5 cents*.
- This yellow box will appear on your screen every 20 seconds whether you are undertaking *Task 2* or *browsing the internet*.

**Earnings formula**

- Your individual earnings correspond to the following sum over the *5* periods:
  - Individual *Task 1 profit* (obtained from clicking the yellow box)

**+**

- - Your share (*10%*) of *Task 2 total contribution* that has been generated by all *10* participants summing up numbers during the experiment.

**Earnings Calculation**

- **EXAMPLE**: In the first period you clicked on the yellow box 45 times and you provided 4 correct answers in *Task 2* while providing one incorrect answer. Also, the other nine participants in the experiment provided a total of 30 correct answers in *Task 2* while providing 5 incorrect answers.
- Your earnings (**“My Period Earnings”**) for that period are equal to:

*Task 1 Profit*: 45×*5 =* **225** *cents*

+*Task 2 Profit*: 10% × (34×*50* - 6×*20*) *=* **158** *cents*

= **My Earnings** (period 1): 225 + 158*=* **383** *cents*
