# Supplementary material for: Cognitive Reflection and the Diligent Worker: An Experimental Study of Millennials
Source: PLoS One. 2015 Nov 6;10(11):e0141243. doi: 10.1371/journal.pone.0141243 (PMC4636387; doi:10.1371/journal.pone.0141243)
Supplement: S2 Text — (DOCX) [file pone.0141243.s002.docx]

**S2 Text. Survey description.**

Descriptive statistics for the variables used in the survey are shown below:

**Demographics:**

**Table A. Gender.**

| Female | 47.7% (n=126) |
| --- | --- |
| Male | 52.3% (n=138) |

**Table B. Religiosity.** *How often do you attend religious services?*

| everyday | 0.0% (n=0) |
| --- | --- |
| more than 1 day per week | 1.9% (n=5) |
| once a week | 15.5% (n=41) |
| once a month | 10.2% (n=27) |
| less than once a month | 24.2% (n=64) |
| never | 47.7% (n=126) |
| Rather not say! | 0.4% (n=1) |

**Table C. Parents’ education.** *Does your Father/Mother have a college degree?*

| None of them | 19.3% (n=51) |
| --- | --- |
| Only Mother | 14.0% (n=37) |
| Only Father | 12.9% (n=34) |
| Both parents | 53.8% (n=142) |

**Table D. High School Math Grade.** *What was your last mathematics grade in High School?*

| A | 53.0% (n=140) |
| --- | --- |
| B | 29.2% (n=77) |
| C | 8.7% (n=23) |
| D or E | 2.7% (n=7) |
| I don’t know | 6.4% (n=17) |

**Table E. Internet habits.** *How many hours per day do you use internet?*

| I don't use it! | 0.4% (n=1) |
| --- | --- |
| less than 1 hour | 1.1% (n=3) |
| between 1 and 3 hours | 22.0% (n=58) |
| between 3 and 5 hours | 37.9% (n=100) |
| between 5 and 7 hours | 26.9% (n=71) |
| more than 7 hours | 11.7% (n=31) |

**Table F. Work.** *How many hours per week do you work for pay?*

| none | 48.5% (n=128) |
| --- | --- |
| between 1 and 5 | 15.5% (n=41) |
| between 6 and 10 | 11.4% (n=30) |
| between 11 and 20 | 17.8% (n=47) |
| between 21 and 40 | 6.4% (n=17) |
| more than 40 | 0.4% (n=1) |

**Table G. Volunteer work.** *How many hours per week do you spend volunteering?*

| none | 59.1% (n=156) |
| --- | --- |
| between 1 and 5 | 33.0% (n=87) |
| between 6 and 10 | 5.7% (n=15) |
| between 11 and 20 | 1.1% (n=3) |
| between 21 and 40 | 0.8% (n=2) |
| more than 40 | 0.4% (n=1) |

**Table H. School.** *In what school are you studying?*

| School of Business and Economics | 23.9% (n=63) |
| --- | --- |
| College of Educational Studies | 4.2% (n=11) |
| College of Film and Media Arts | 27.7% (n=73) |
| College of Humanities and Social Sciences | 20.5% (n=54) |
| School of Law | 1.1% (n=3) |
| College of Performing Arts | 6.8% (n=18) |
| College of Science | 15.9% (n=42) |

**Cognitive measures:**

**Table I. Adding skills.**

| Average | 28.9 |
| --- | --- |
| Median | 27.0 |
| Std. Dev. | 9.7 |
| Min | 10 |
| Max | 72 |

**Table J. Cognitive Reflection Test (CRT).** Frederick (2005).

| Score | Frequency |
| --- | --- |
| 0 | 33.0% (n=87) |
| 1 | 30.7% (n=81) |
| 2 | 17.4% (n=46) |
| 3 | 18.9% (n=50) |

The CRT score is based on the answers to the following three questions:

1. *A bat and a ball cost $1.10 in total. The bat costs $1.00 more than the ball. How much does the ball cost?*
2. *If it takes 5 machines 5 minutes to make 5 widgets, how long would it take 100 machines to make 100 widgets?*
3. *In a lake, there is a patch of lily pads. Every day, the patch doubles in size. If it takes 48 days for the patch to cover the entire lake, how long would it take for the patch to cover half of the lake?*

**Table K. Grade Point Average (GPA).**

| less than 2 | 0.4% (n=1) |
| --- | --- |
| between 2 and 2.5 | 1.1% (n=3) |
| between 2.5 and 3 | 9.1% (n=24) |
| between 3 and 3.5 | 39.4% (n=104) |
| more than 3.5 | 48.1% (n=127) |
| I don't know | 1.9% (n=5) |

**Table L. Scholastic Aptitude Test (SAT).**

| less than 1000 | 0.0% (n=0) |
| --- | --- |
| between 1001 and 1250 | 1.1% (n=3) |
| between 1251 and 1500 | 5.3% (n=14) |
| between 1501 and 1800 | 21.2% (n=56) |
| between 1801 and 2000 | 29.2% (n=77) |
| between 2001 and 2200 | 21.6% (n=57) |
| between 2201 and 2400 | 3.8% (n=10) |
| I don't know | 17.8% (n=47) |

**Personality:**

**Table M. The Big Five Inventory.** John, Donahue and Kentle (1991) and John, Naumann and Soto (2008)

| Variable | Α | Mean | Median | Std. Dev. | Min | Max |
| --- | --- | --- | --- | --- | --- | --- |
| Openness | .80 | 38.6 | 39.0 | 5.7 | 21.0 | 50.0 |
| Conscientiousness | .80 | 33.7 | 34.0 | 5.2 | 19.0 | 44.0 |
| Extraversion | .87 | 27.4 | 28.0 | 5.8 | 14.0 | 40.0 |
| Agreeableness | .78 | 34.6 | 35.0 | 5.0 | 15.0 | 45.0 |
| Neuroticism | .81 | 21.3 | 21.0 | 5.5 | 9.0 | 37.0 |

| **TABLE N.** Pairwise correlations of Cognitive Measures and Personality Traits. | | | | |
| --- | --- | --- | --- | --- |
|  | CRT | GPA | SAT | Math Skills |
| Openness | 0.1231** | 0.0852 | 0.1561** | -0.0187 |
| Conscientiousness | -0.1195* | 0.2423**** | 0.0245 | 0.0773 |
| Extraversion | -0.0687 | -0.136** | -0.0435 | 0.0069 |
| Agreeableness | -0.2005*** | -0.0652 | -0.1137* | -0.0688 |
| Neuroticism | 0.0554 | -0.0897 | 0.0161 | -0.1231** |
| *p -value<.10, ** p-value<.05, *** p-value<.01, and **** p-value<.001 | | | | |

| **TABLE O.** Pairwise correlations of Personality Traits. | | | | | |
| --- | --- | --- | --- | --- | --- |
|  | [1] | [2] | [3] | [4] | [5] |
| 1. Openness | 1 |  |  |  |  |
| 2. Conscientiousness | -0.0105 | 1 |  |  |  |
| 3. Extraversion | 0.1482** | 0.176*** | 1 |  |  |
| 4. Agreeableness | 0.0289 | 0.1365** | 0.0938 | 1 |  |
| 5. Neuroticism | -0.0018 | -0.1435** | -0.139** | -0.2472**** | 1 |
| *p -value<.10, ** p-value<.05, *** p-value<.01, and **** p-value<.001 | | | | | |
