# Supplementary material for: Cognitive Reflection and the Diligent Worker: An Experimental Study of Millennials
Source: PLoS One. 2015 Nov 6;10(11):e0141243. doi: 10.1371/journal.pone.0141243 (PMC4636387; doi:10.1371/journal.pone.0141243)
Supplement: S3 Text — (DOCX) [file pone.0141243.s003.docx]

**S3 Text. Regression results.**

We report OLS regressions with robust standard errors and clusters at the session level for *productivity* (**Table A**), *accuracy* (**Table B**) and *cyberloafing* (**Table C**).

| **TABLE A.** OLS regressions with robust standard errors for *Productivity*. | | | | | |
| --- | --- | --- | --- | --- | --- |
| Independent variables |  |  |  |  |  |
| Constant | 1.74  (13.09) | 27.47*  (14.75) | 23.82  (16.24) | 32.44*  (16.37) | 2.54  (15.94) |
| Cognitive measures |  |  |  |  |  |
| Adding skills | 0.71****  (0.13) | - | - | - | 0.66****  (0.14) |
| CRT | - | 3.05****  (0.76) | - | - | 1.72**  (0.79) |
| SAT | - | - | 2.66**  (1.11) | - | 1.26  (0.97) |
| GPA | - | - | - | -0.25  (1.87) | -0.58  (1.84) |
| Personality traits (Big Five) |  |  |  |  |  |
| Openness | -0.05  (0.12) | -0.18  (0.15) | -0.14  (0.16) | -0.09  (0.16) | -0.12  (0.14) |
| Conscientiousness | -0.07  (0.13) | 0.10  (0.16) | 0.00  (0.20) | 0.01  (0.16) | -0.07  (0.18) |
| Extraversion | 0.10  (0.14) | 0.06  (0.15) | 0.16  (0.14) | 0.07  (0.16) | 0.15  (0.14) |
| Agreeableness | 0.01  (0.17) | 0.04  (0.19) | -0.06  (0.19) | -0.06  (0.19) | 0.01  (0.19) |
| Neuroticism | -0.05  (0.21) | -0.19  (0.23) | -0.16  (0.25) | -0.13  (0.24) | -0.14  (0.22) |
| Organizational dummies |  |  |  |  |  |
| Incentives | 9.29****  (2.67) | 6.23**  (3.09) | 6.45*  (3.27) | 6.29*  (3.24) | 9.37***  (3.19) |
| Chat | 4.70*  (2.45) | 3.05  (2.92) | 2.06  (3.36) | 2.94  (3.05) | 4.06  (2.83) |
| Watch | 3.52  (2.69) | 2.68  (3.24) | 4.54  (3.49) | 3.03  (3.38) | 4.79  (2.91) |
| Demographics |  |  |  |  |  |
| Female | 0.68  (1.70) | -1.84  (1.82) | -4.19**  (1.69) | -3.56*  (1.80) | 1.26  (1.80) |
| Religiosity | 0.22  (0.72) | 0.06  (0.77) | 0.66  (0.88) | 0.07  (0.79) | 0.46  (0.86) |
| Parents’ education | 0.25  (1.04) | -0.66  (1.07) | -1.11  (1.37) | -0.22  (1.18) | -0.69  (1.20) |
| High-School Math grade | 0.32  (0.98) | -0.22  (1.20) | -0.31  (1.43) | 0.57  (1.32) | -0.17  (1.43) |
| Internet habits | 0.79  (0.90) | 0.31  (0.98) | 0.40  (1.05) | 0.01  (0.96) | 1.12  (1.06) |
| Work experience | 0.73  (0.66) | 0.72  (0.73) | 0.60  (0.91) | 0.62  (0.81) | 0.67  (0.72) |
| Volunteering | 0.11  (0.89) | 0.57  (0.93) | -0.02  (1.09) | -0.10  (1.01) | 0.52  (1.04) |
| School*:[Omitted: Film & Media Arts]* |  |  |  |  |  |
| Business & Economics | 0.10  (2.16) | 3.31  (2.70) | 3.31  (2.91) | 3.55  (2.86) | -0.60  (2.42) |
| Educational Studies | -2.98  (2.37) | -2.93  (3.21) | -3.52  (3.79) | -3.36  (3.43) | -2.85  (2.89) |
| Humanities & Social Sciences | 0.19  (2.01) | 0.58  (2.30) | 0.65  (2.33) | 0.91  (2.20) | -0.31  (2.31) |
| Law | 12.67**  (5.49) | 9.59*  (4.83) | 13.10***  (4.37) | 12.99***  (3.90) | 10.53  (4.70) |
| Performing Arts | -1.10  (4.01) | 3.28  (4.43) | 5.69  (5.81) | 2.91  (4.98) | 1.19  (4.66) |
| Science | 0.28  (2.57) | 0.08  (2.61) | -0.25  (3.14) | 1.91  (2.76) | -2.79  (3.42) |
| Number of observations | *n* = 246 | *n* = 246 | *n* = 205 | *n* = 243 | *n* = 204 |
| R² | R²=0.27 | R²=0.13 | R²=0.13 | R²=0.08 | R²=0.30 |
| F(22,47)  F(25,47) | F=9.28**** | F=7.83**** | F=3.91**** | F=8.22**** | F=27.13**** |
| *p -value<.10, ** p-value<.05, *** p-value<.01, and **** p-value<.001 | | | | | |

| **TABLE B.** OLS regressions with robust standard errors for *Accuracy*. | | | | | |
| --- | --- | --- | --- | --- | --- |
| Independent variables |  |  |  |  |  |
| Constant | 22.22  (20.13) | 40.46*  (21.37) | 1.72  (23.32) | 43.47*  (24.52) | -11.96  (19.55) |
| Cognitive measures |  |  |  |  |  |
| Adding skills | 0.58***  (0.18) | - | - | - | 0.48**  (0.20) |
| CRT | - | 7.07****  (1.61) | - | - | 5.08***  (1.87) |
| SAT | - | - | 3.73*  (2.21) | - | 1.62  (2.18) |
| GPA | - | - | - | -1.70  (2.55) | -0.73  (2.80) |
| Personality traits (Big Five) |  |  |  |  |  |
| Openness | 0.30  (0.37) | 0.02  (0.39) | 0.54  (0.37) | 0.31  (0.38) | 0.45  (0.38) |
| Conscientiousness | 0.23  (0.32) | 0.47  (0.31) | 0.55*  (0.31) | 0.46  (0.32) | 0.56*  (0.31) |
| Extraversion | -0.28  (0.35) | -0.30  (0.33) | -0.26  (0.36) | -0.29  (0.35) | -0.28  (0.37) |
| Agreeableness | -0.06  (0.32) | 0.07  (0.33) | 0.22  (0.37) | -0.09  (0.34) | 0.33  (0.37) |
| Neuroticism | 0.00  (0.31) | -0.21  (0.30) | -0.17  (0.32) | -0.09  (0.32) | -0.25  (0.30) |
| Organizational dummies |  |  |  |  |  |
| Incentives | 8.70  (7.43) | 5.69  (7.39) | 5.09  (9.66) | 5.94  (7.86) | 7.04  (8.93) |
| Chat | 9.23*  (5.46) | 8.05  (5.30) | 8.95  (5.76) | 7.48  (5.82) | 10.72**  (5.30) |
| Watch | 4.87  (5.52) | 3.75  (5.00) | 6.14  (5.85) | 4.29  (5.75) | 5.87  (5.45) |
| Demographics |  |  |  |  |  |
| Female | 3.67  (4.55) | 4.34  (3.72) | -0.10  (4.21) | -0.46  (4.00) | 6.58  (4.74) |
| Religiosity | 1.39  (1.64) | 1.28  (1.63) | 3.34**  (1.40) | 1.65  (1.70) | 3.12**  (1.49) |
| Parents’ education | 2.03  (1.96) | 0.58  (1.72) | -1.10  (2.29) | 1.34  (1.91) | -0.93  (2.21) |
| High-School Math grade | 1.01  (2.01) | -0.58  (2.28) | 0.68  (2.58) | 1.30  (2.17) | 0.52  (2.66) |
| Internet habits | 1.11  (1.52) | 1.03  (1.51) | 1.01  (1.44) | 0.99  (1.52) | 1.75  (1.51) |
| Work experience | 2.33**  (1.06) | 2.39**  (0.98) | 2.14*  (1.18) | 2.30**  (1.12) | 2.33**  (1.08) |
| Volunteering | -2.67  (2.49) | -1.29  (2.63) | -2.09  (2.42) | -2.96  (2.68) | -1.00  (2.55) |
| School*:[Omitted: Film & Media Arts]* |  |  |  |  |  |
| Business & Economics | -2.24  (4.44) | -0.28  (4.33) | -0.26  (4.96) | 0.03  (4.78) | -3.56  (5.02) |
| Educational Studies | -22.26**  (10.96) | -21.73*  (11.36) | -17.27*  (9.91) | -22.92*  (11.62) | -16.08*  (9.53) |
| Humanities & Social Sciences | -8.33*  (4.75) | -8.17*  (4.44) | -10.99**  (5.48) | -9.14*  (4.86) | -12.01  (5.50) |
| Law | 6.58  (18.51) | -1.60  (10.10) | 12.31  (10.91) | 5.86  (14.92) | 6.14  (10.06) |
| Performing Arts | -15.04*  (8.12) | -10.99  (7.09) | -10.67  (8.86) | -11.95  (8.16) | -13.93  (9.10) |
| Science | -4.43  (6.01) | -7.57  (6.28) | -1.05  (6.91) | -3.61  (6.22) | -5.15  (7.15) |
| Number of observations | *n* = 244 | *n* = 244 | *n* = 204 | *n* = 241 | *n* = 203 |
| R² | R²=0.13 | R²=0.16 | R²=0.14 | R²=0.10 | R²=0.20 |
| F(22,47)  F(25,47) | F=4.33**** | F=4.38**** | F=4.06**** | F=1.99** | F=9.81**** |
| *p -value<.10, ** p-value<.05, *** p-value<.01, and **** p-value<.001 | | | | | |

| **TABLE C.** OLS regressions with robust standard errors for *Cyberloafing*. | | | | | |
| --- | --- | --- | --- | --- | --- |
| Independent variables |  |  |  |  |  |
| Constant | 51.85***  (16.42) | 52.56****  (15.56) | 42.10**  (17.07) | 54.52****  (15.55) | 43.33**  (18.57) |
| Cognitive measures |  |  |  |  |  |
| Adding skills | -0.02  (0.10) | - | - | - | 0.07  (0.10) |
| CRT | - | -2.32**  (0.94) | - | - | -2.34**  (1.11) |
| SAT | - | - | -1.22  (1.15) | - | -0.47  (1.13) |
| GPA | - | - | - | -0.35  (1.87) | -1.21  (2.19) |
| Personality traits (Big Five) |  |  |  |  |  |
| Openness | -0.19  (0.19) | -0.11  (0.19) | -0.08  (0.20) | -0.17  (0.18) | 0.00  (0.21) |
| Conscientiousness | -0.26  (0.16) | -0.32*  (0.16) | -0.24  (0.17) | -0.28*  (0.16) | -0.24  (0.18) |
| Extraversion | 0.12  (0.23) | 0.13  (0.23) | 0.08  (0.23) | 0.12  (0.23) | 0.06  (0.24) |
| Agreeableness | -0.23  (0.26) | -0.28  (0.27) | -0.18  (0.28) | -0.28  (0.26) | -0.26  (0.28) |
| Neuroticism | 0.05  (0.27) | 0.10  (0.26) | 0.11  (0.28) | 0.05  (0.27) | 0.15  (0.27) |
| Organizational dummies |  |  |  |  |  |
| Incentives | -17.26****  (4.59) | -16.97****  (4.51) | -16.60***  (5.19) | -17.68****  (4.81) | -16.30***  (5.25) |
| Chat | -9.12**  (3.61) | -9.24**  (3.52) | -6.75*  (3.81) | -9.15**  (3.77) | -6.86*  (3.97) |
| Watch | -8.73***  (3.15) | -8.47***  (3.06) | -10.81***  (3.64) | -8.92***  (3.23) | -10.86***  (3.67) |
| Demographics |  |  |  |  |  |
| Female | -3.94  (2.61) | -5.21**  (2.30) | -3.71*  (2.21) | -3.67  (2.30) | -4.87*  (2.56) |
| Religiosity | -0.98  (0.90) | -0.95  (0.88) | -1.11  (0.94) | -1.03  (0.90) | -1.07  (0.91) |
| Parents’ education | -1.37  (1.43) | -1.01  (1.38) | -0.17  (1.60) | -1.24  (1.40) | -0.04  (1.56) |
| High-School Math grade | 0.11  (1.25) | 0.65  (1.20) | 1.35  (1.37) | 0.03  (1.39) | 1.69  (1.42) |
| Internet habits | -1.52  (1.23) | -1.65  (1.25) | -1.22  (1.35) | -1.53  (1.25) | -1.13  (1.46) |
| Work experience | -0.02  (0.87) | -0.08  (0.84) | 0.64  (1.07) | 0.00  (0.89) | 0.58  (1.07) |
| Volunteering | -0.64  (1.15) | -1.13  (1.15) | -1.30  (1.20) | -0.70  (1.25) | -1.81  (1.22) |
| School*:[Omitted: Film & Media Arts]* |  |  |  |  |  |
| Business & Economics | 1.41  (2.97) | 1.56  (3.02) | 2.82  (3.30) | 1.09  (2.79) | 2.10  (3.27) |
| Educational Studies | 2.38  (4.63) | 2.09  (4.43) | 4.40  (6.31) | 2.51  (4.72) | 3.83  (6.32) |
| Humanities & Social Sciences | 4.32  (2.99) | 4.47  (3.07) | 4.54  (3.32) | 4.28  (2.93) | 4.17  (3.43) |
| Law | -16.38***  (5.68) | -13.59***  (4.42) | -13.57**  (5.91) | -16.87***  (5.22) | -12.75  (5.63) |
| Performing Arts | 5.73  (4.61) | 5.34  (4.16) | 3.81  (5.79) | 5.30  (4.65) | 2.88  (5.77) |
| Science | 3.09  (3.96) | 4.46  (3.99) | 6.97  (5.02) | 2.85  (3.82) | 7.54  (4.94) |
| Number of observations | *n* = 246 | *n* = 246 | *n* = 205 | *n* = 243 | *n* = 204 |
| R² | R²=0.17 | R²=0.19 | R²=0.19 | R²=0.18 | R²=0.21 |
| F(22,47)  F(25,47) | F=4.89**** | F=6.29**** | F=8.40**** | F=5.22**** | F=5.16**** |
| *p -value<.10, ** p-value<.05, *** p-value<.01, and **** p-value<.001 | | | | | |
