# Supplementary material for: Cognitive Reflection and the Diligent Worker: An Experimental Study of Millennials
Source: PLoS One. 2015 Nov 6;10(11):e0141243. doi: 10.1371/journal.pone.0141243 (PMC4636387; doi:10.1371/journal.pone.0141243)
Supplement: S4 Text — (DOCX) [file pone.0141243.s004.docx]

**S4 Text. Descriptive statistics and standard tests**

We report descriptive statistics and standard tests for the effect of CRT scores on *productivity* (**Table A**), *accuracy* (**Table B**) and *cyberloafing* (**Table C**).

**TABLE A.** Descriptive statistics and standard tests for the effect of CRT scores on productivity.

Average [median] (number of observations)

|  |  | CRT |  |  | p-values T-Test |
| --- | --- | --- | --- | --- | --- |
| Treatment | 0 | 1 | 2 | 3 | (Ranksum Test)^+^ |
| 1 (n=47) | 28.15 [11.27]  (n=13) | 32.85 [12.39]  (n=20) | 34.25 [17.09]  (n=8) | 44.67 [15.42]  (n=6) | **0.080**  (0.176) |
| 2 (n=41) | 30.00 [8.76]  (n=11) | 31.29 [13.96]  (n=17) | 40.71 [3.73]  (n=7) | 35.33 [16.79]  (n=6) | **0.068**  **(0.036)** |
| 3 (n=38) | 28.73 [10.14]  (n=15) | 31.63 [5.45]  (n=8) | 40.90 [24.04]  (n=10) | 40.00 [8.94]  (n=5) | **0.026**  **(0.036)** |
| 4 (n=30) | 20.75 [13.55]  (n=8) | 21.33 [11.19]  (n=12) | 23.33 [13.32]  (n=3) | 27.29 [16.01]  (n=7) | 0.321  (0.467) |
| 5 (n=37) | 29.64 [11.21]  (n=11) | 39.82 [12.18]  (n=11) | 34.13 [8.43]  (n=8) | 31.29 [12.85]  (n=7) | >0.50  (0.278) |
| 6 (n=38) | 32.23 [8.21]  (n=13) | 28.57 [18.83]  (n=7) | 29.33 [7.63]  (n=6) | 42.58 [22.24]  (n=12) | 0.178  (>0.50) |
| 7 (n=33) | 28.06 [8.14]  (n=16) | 31.50 [5.96]  (n=6) | 17.75 [13.18]  (n=4) | 33.86 [11.54]  (n=7) | >0.50  (>0.50) |
| All treatments  (n=264) | 28.59 [10.10]  (n=87) | 31.17 [12.99]  (n=81) | 33.87 [15.86]  (n=46) | 36.76 [16.55]  (n=50) | **0.001**  **(0.007)** |

^+^ We compare *production* between below- (0 or 1) and above-median (2 or 3) CRT subjects.

**TABLE B.** Descriptive statistics and standard tests for the effect of CRT scores on accuracy.

Average [median] (number of observations)

|  |  | CRT |  |  | p-values T-Test |
| --- | --- | --- | --- | --- | --- |
| Treatment | 0 | 1 | 2 | 3 | (Ranksum Test)^+^ |
| 1 (n=47) | 54.33 [31.81]  (n=13) | 64.54 [30.64]  (n=20) | 78.09 [21.39]  (n=8) | 88.40 [4.10]  (n=6) | **0.016**  **(0.002)** |
| 2 (n=41) | 69.34[14.61]  (n=11) | 66.94 [20.66]  (n=17) | 74.65 [11.89]  (n=7) | 80.19 [17.61]  (n=6) | 0.113  **(0.090)** |
| 3 (n=38) | 60.49 [29.59]  (n=15) | 70.78 [24.75]  (n=8) | 79.52 [9.53]  (n=10) | 83.58 [3.42]  (n=5) | **0.029**  **(0.028)** |
| 4 (n=30) | 44.67 [40.22]  (n=8) | 48.79 [32.72]  (n=12) | 90.35 [8.44]  (n=3) | 67.95 [30.63]  (n=7) | **0.038**  **(0.013)** |
| 5 (n=37) | 45.91 [37.72]  (n=11) | 74.71 [10.56]  (n=11) | 78.27 [9.96]  (n=8) | 68.02 [31.63]  (n=7) | 0.165  (0.129) |
| 6 (n=38) | 64.41 [32.45]  (n=13) | 47.76 [37.19]  (n=6) | 69.28 [35.56]  (n=6) | 82.45 [10.55]  (n=12) | **0.053**  **(0.067)** |
| 7 (n=33) | 72.22 [24.95]  (n=16) | 63.83 [17.76]  (n=6) | 74.42 [6.00]  (n=3) | 73.92 [16.28]  (n=7) | >0.050  (>0.50) |
| All treatments  (n=262) | 60.13 [30.90]  (n=87) | 63.40 [26.73]  (n=80) | 77.30 [17.28]  (n=45) | 77.76 [19.54]  (n=50) | **<0.0001**  **(<0.0001)** |

^+^ We compare *accuracy* between below- (0 or 1) and above-median (2 or 3) CRT subjects.

**TABLE C.** Descriptive statistics and standard tests for the effect of CRT scores on *cyberloafing*.

Average [std. dev] (number of observations)

|  |  | CRT |  |  | p-values T-Test |
| --- | --- | --- | --- | --- | --- |
| Treatment | 0 | 1 | 2 | 3 | (Ranksum Test)^+^ |
| 1 (n=47) | 2.44 [3.33]  (n=13) | 9.11 [18.39]  (n=20) | 9.39 [22.34]  (n=8) | 1.04 [0.80]  (n=6) | >0.50  **0.028** |
| 2 (n=41) | 5.00 [6.62]  (n=11) | 9.39 [19.23]  (n=17) | 8.45 [10.02]  (n=7) | 7.87 [15.35]  (n=6) | >0.50  (>0.50) |
| 3 (n=38) | 9.65 [14.48]  (n=15) | 10.18 [7.41]  (n=8) | 9.64 [10.92]  (n=10) | 11.15 [12.22]  (n=5) | >0.50  (>0.50) |
| 4 (n=30) | 34.83 [28.99]  (n=8) | 29.10 [23.98]  (n=12) | 25.17 [32.51]  (n=3) | 20.76 [28.24]  (n=7) | 0.368  (0.291) |
| 5 (n=37) | 12.89 [19.71]  (n=11) | 6.89 [12.16]  (n=11) | 3.00 [3.98]  (n=8) | 4.02 [5.33]  (n=7) | 0.147  (0.421) |
| 6 (n=38) | 5.52 [8.17]  (n=13) | 22.07 [26.86]  (n=7) | 1.50 [1.85]  (n=6) | 6.10 [11.80]  (n=12) | 0.172  **(0.044)** |
| 7 (n=33) | 6.98 [8.08]  (n=16) | 3.63 [4.44]  (n=6) | 24.62 [36.71]  (n=4) | 8.56 [13.44]  (n=7) | 0.140  (0.40) |
| All treatments  (n=264) | 9.60 [15.81]  (n=87) | 12.65 [19.42]  (n=81) | 9.52 [17.53]  (n=46) | 8.31 [14.97]  (n=50) | 0.3208  **(0.066)** |

^+^ We compare *cyberloafing* between below- (0 or 1) and above-median (2 or 3) CRT subjects.
